# Analogical thinking modifiability and math processing strategy

**DOI:** 10.3389/fpsyg.2024.1339591

**Published:** 2024-02-28

**Authors:** David Tzuriel

**Affiliations:** ^1^Talpiot Academic College, Graduate Program of Learning Disabilities, Holon, Israel; ^2^Faculty of Education, Bar-Ilan University, Ramat Gan, Israel

**Keywords:** analogical thinking, dynamic assessment, mathematical performance, mediated learning, executive functions, mathematical processing strategy

## Abstract

A sample of 48 children in Grade 2 was randomly assigned to an experimental (*n* = 24) and a control group (*n* = 24). Both groups were administered the Analogical Modifiability Puzzle Test (AMPT) and Math Accuracy and Processing Strategy (MAPS) test before and after a teaching phase of the AMPT. The MAPS test includes scores for Accuracy, Processing Strategy, and a Math-Total. The findings reveal significant treatment x time interactions for AMPT (near-transfer) and MAPS (far-transfer) scores. Hierarchical regression analysis showed that AMPT post-teaching score added significantly to Math Total. The findings indicate that Math Accuracy and Math Processing Strategies are affected by mediation for analogical thinking and that modifiability of analogical thinking significantly predicts Math-Total.

## Introduction

Analogical thinking is a fundamental cognitive operation that plays a role in executing many academic and daily life skills (Gentner et al., 2003), especially skills related to STEM domains (Alexander, 2017). Analogical thinking is a cognitive operation that helps individuals to represent information and objects as the structures of relationships. These structures can be compared and united in innovative ways depending on the contextual objectives (e.g., Gentner et al., 2003; Holyoak, 2004). Learning by analogy involves flexible conceptual learning and problem-solving (Goswami, 1992), especially in mathematics (Gelman, 2000). It was reported, for example, that, when US high-school students engage in solving math problems, they are found to be retrieving procedures rather than perceiving the relationship and similarities between concepts and strategies. In other words, their processing strategies for solving math problems lack analogical thinking (Stigler et al., 2010; Givvin et al., 2011).

Analogical thinking was reported to be central to mathematical learning and thinking (e.g., Richland and McDonough, 2010; Vamvakoussi and Vosniadou, 2012; Vamvakoussi, 2017, 2019; Zhao et al., 2021). Most studies on the use of analogy in math education refer to within-domain (i.e., mathematics) transfer of concepts such as the complex relation between geometry and arithmetic (Dantzig, 2005). The objectives of this study were (a) to examine the transfer of analogical thinking in a figural modality of a cognitive non-mathematical domain to math performance and math processing strategies and (b) to find the prediction of improvement in math performance by analogical thinking modifiability. The transfer is examined after a short-term teaching of figural analogies within a dynamic assessment (DA) procedure (Tzuriel and George, 2009; Tzuriel and Shamir, 2010; Tzuriel, 2021a). It should be noted that the teaching of figural analogies involves, in addition to transformative rules of analogical thinking, mediation of executive functions such as control of impulsivity, systematic exploratory behavior, and working memory (Feuerstein et al., 2002; Baddeley, 2012).

### Analogical thinking and mathematical skills

Analogical thinking and math achievements have been found to be intimately related both at theoretical and practical levels (e.g., Bassok and Holyoak, 1989; Goswami, 1992; Gelman, 2000; Resing, 2000; English, 2004; Richland and Simms, 2015). Learning by analogy enables the use of common concepts and strategies between mathematical representations and enhances the understanding of new mathematical constructs and synchronization between mathematical components (Gelman, 2000; Gonzales et al., 2001; National Mathematics Advisory Panel, 2008). Using analogies in a learning process involves finding a systematic correspondence or “mapping” two domains: an acquainted *source* analog and a novel *target*, which is concrete or abstract (Sternberg and Nigro, 1980). For example, in mathematics, the acquainted source could refer to solving inequalities, and the projected novel target is solving equations (Gick and Holyoak, 1987). Many researchers agree that mathematical reasoning involves projecting (transfer) the abstract mathematical relations (e.g., proportion, equality, integral) into different contexts (e.g., Gelman, 2000; Gentner et al., 2003). Several researchers suggest the concepts of *conceptual metaphors* (e.g., Núñez and Lakoff, 2005), *schema construction* (Gick and Holyoak, 1983), and *bridging analogies* (e.g., Clement, 1993, 2013) to refer to cross-domain mappings (i.e., transfer concept from one domain to another).

In one of the intriguing cross-cultural studies, Richland et al. (2007) compared the effects of the use of analogies in math teaching among teachers in the United States, Hong Kong (HK) and Japan. The reasons for the comparison were based on two main earlier findings: (a) students in HK and Japan consistently outperformed students from the United States on the Trends in International Mathematics and Science Study (TIMSS) international achievement tests (e.g., Mullis et al., 2004) and (b) different classroom instructional practices were observed between the teachers of the USA and two Asian countries. The goal of Richland et al.'s (2007) study was to ascertain what cognitive and memory factors may explain why US students achieve lower scores in international math tests. The findings indicated that the US teachers provide the same number of analogies as the Asian teachers; however, they provide much less *cognitive processing support* that facilitates relational learning. For example, Asian teachers used far more gestures and more than twice as many prompts of mental and visual imagery (in HK) and spatial supports for comparison (in HK and Japan) than US teachers. The authors interpreted these findings by attributing diverse cultural orientations to analogical reasoning. Teachers in Japan and HK are found to be more attentive to processing demands using analogical reasoning than US teachers. The processing demands include memory search to understand the analogy source, accessibility to a visual image that supports working memory (WM) during the construction of the analogy, and reliance on cues that guide the analogy process.

Richland and McDonough (2010) showed that reducing the processing load during instructional analogies facilitated learning and relational reasoning. These authors showed, in a sample of undergraduate students, that supporting comparative reasoning with cues facilitated the students' ability to discriminate between relevant analogs at a test given later. Similarly, Rittle-Johnson and Star (2007) compared two teaching strategies: simultaneous visual–spatial representations of analogs (i.e., problems solved in two ways were presented simultaneously and an explicit requirement to compare them) and solutions presented sequentially one after another. The authors reported that simultaneous representation enhanced the students' procedural and conceptual understanding and that their retention of the procedures, were better transferred to novel analogy problems.

### The dynamic assessment of cognitive modifiability

The term dynamic assessment (DA) is defined as “an assessment of thinking, perception, learning, and problem-solving by an active teaching process aimed at modifying cognitive function” (Tzuriel, 2021a, p. 69). The main goal of DA is to assess the *cognitive modifiability* and learning potential using mediated learning strategies. Cognitive modifiability affords an indication of learning potential. However, the learning potential may be actualized depending on the provision of appropriate intervention aimed at the actualization of the individual's abilities (Tzuriel, 2001, 2002, 2021a,b; Feuerstein et al., 2002). The idea upon which DA is based is that measures of cognitive modifiability are intimately related to mediational processes by which the child learns how to process information far more than they are to the standardized measures of intelligence. In other words, the strategies of mediation used within the DA procedure are similar much more to the learning processes in an academic domain and other life situations compared to standardized testing methods and, therefore, provide better indications about future changes in cognitive structures.

In a previous study with the *Analogical Thinking Modifiability Test* (AMPT, Tzuriel, 2022), the goals were (a) to investigate whether mediation with Construction analogies is more powerful than mediation with Closed analogies to improve analogical thinking. In Construction analogies, the child must construct the answer using tactile colored puzzle-like parts and a plate with four (2 × 2) “windows” in which the puzzle-like design is created. The solution is done in a sequential process that does not require the activation of WM. In the Closed analogies version, the child is presented with three parts of the analogy (A:B::C:__) and is asked to choose the correct answer from six designs presented at the bottom of the page. (b) The second goal was to assess whether the mediation of solving analogies improves spatial WM, as measured by the *Children's Spatial Working Memory* test (CSWM, Tzuriel, 2021c). More specifically, the question was which type of mediation—mediation of Construction or mediation of Closed analogies—transfers better to mathematics? (c) The last goal was to explore the correlation between analogical thinking and WM. The findings indicated clearly that mediation with Construction analogies was more effective in enhancing analogical thinking than mediation with Closed analogies and that both groups improved their spatial WM. The reason for the superiority of Construction analogies is that the format of Construction analogies contains imminent characteristics of self-regulation and systematic sequential and progressive activity, which reduce cognitive load and WM requirements. In Closed analogies, on the other hand, the examinee is required to maintain the solution in his/her *memory sketchpad* (Baddeley, 2012) during the search for the correct answer and sorting out distractors. The explanation was further supported by the differential correlation between AMPT performance time and CSWM. The correlations found for Construction analogies were 0.14 and 0.22 (*p* > 0.05), for pre-teaching and post-teaching, respectively. The correlations for Closed analogies were 0.47 and 0.47 (*p* < 0.001), for pre- and post-teaching, respectively. It seems that, in Closed analogies, a longer performance time helps individuals to cope with the task's requirement for WM. In contrast, Construction analogies do not require the activation of WM and hence its low correlation with spatial WM. The findings of the previous study raised the question of whether the improvement in analogical thinking was caused by the mediation strategies *per se* or the mere exposure and practice of the analogy problems. This question is explored further in the current study by contrasting a group that received the mediation of analogical thinking with a group that practiced the same teaching items without mediation. I chose to focus on Closed analogies, which are found to be more related to WM than to Construction analogies. Furthermore, in the current study, the focus was extended to include the far-transfer effects of mediation of analogical thinking on math performance and math processing strategies conceived to be related to the executive functions of WM (e.g., Richland et al., 2007).

In the current study, the measurement/research version of DA was applied (Tzuriel, 1997, 2001, 2020, 2021a). This version includes the preliminary, pre-teaching, teaching, and post-teaching phases; the last three phases consist of parallel items. In the preliminary phase, the child is acquainted with the dimensions of the tasks and cognitive strategies to solve the problems (e.g., referring to task dimensions). The focus of the teaching phase is on processing the analogical rules of transformation, efficient strategies (e.g., use of one dimension at a time), applying verbal anticipation of a solution, and making efficient use of executive functions such as self-regulation and WM. Cognitive modifiability is measured quantitatively and is indicated by the pre- to post-teaching improvement. Previous DA findings revealed that children in kindergarten and first years of primary school could solve complex analogies after a teaching phase than what might have been expected developmentally of children their age (Tzuriel and Klein, 1985; Tzuriel, 2000, 2001, 2020, 2021a). Children in Grade 2 were randomly assigned to the experimental (EX) and control (CN) groups. Both groups received the *AMPT* and the *Math Accuracy and Processing Strategy (MAPS)* Test (see Measures). Children in the EX group received a teaching phase of analogies using mediation strategies by trained experimenters. Children in the CN group solved the same analogies without teaching. Both groups were evaluated again after the intervention with the AMPT and MAPS test.

### Hypotheses

1. Children in the EX group who received the mediation of analogical thinking will demonstrate higher improvement on AMPT analogies from pre- to post-teaching than children in the CN group.
2. Children in the EX group who received the mediation of analogical thinking will demonstrate higher improvement in the MAPS test than children in the CN group.
3. Cognitive modifiability of analogical thinking will significantly predict the modifiability in MAPS scores.

## Method

### Participants

The participants were 48 children (25 boys and 23 girls) in Grade 2 who were randomly selected from six classes from six different schools in the central region of Israel. Students with learning problems (e.g., learning disability, attention-deficit hyperactivity disorder, autism spectrum disorder) were excluded from the sample. The children were randomly assigned to the EX (*n* =24) and CN (*n*= 24) groups. The mean age of children in the EX group was 87.96 months (SD = 2.90) and in the CN group was 89.33 months (SD = 3.17). Both groups were similar on variables of age, t_(46)_ = −1.56 2, *p* > 0.05, gender distribution, χ^2^ = 0.08 *p* > 0.05, occupation level of fathers, χ^2^ = 0.22 *p* > 0.05, and of mothers χ^2^ = 0.93 *p* > 0.05 (on a 1–5 scale). The trainers were graduate students in a Learning Disabilities Program of a college for teachers with 4–15 years of teaching experience. All trainers received 8 h of training prior to the administration of tests and mediation strategies. The training included proficiency in test administration, specific mediation strategies of the analogies in the teaching phase, practice of administration with two children prior to the start of the study, and accurate recording and scoring.

### Measures

#### The analogical modifiability puzzle test

**Figure 1:**
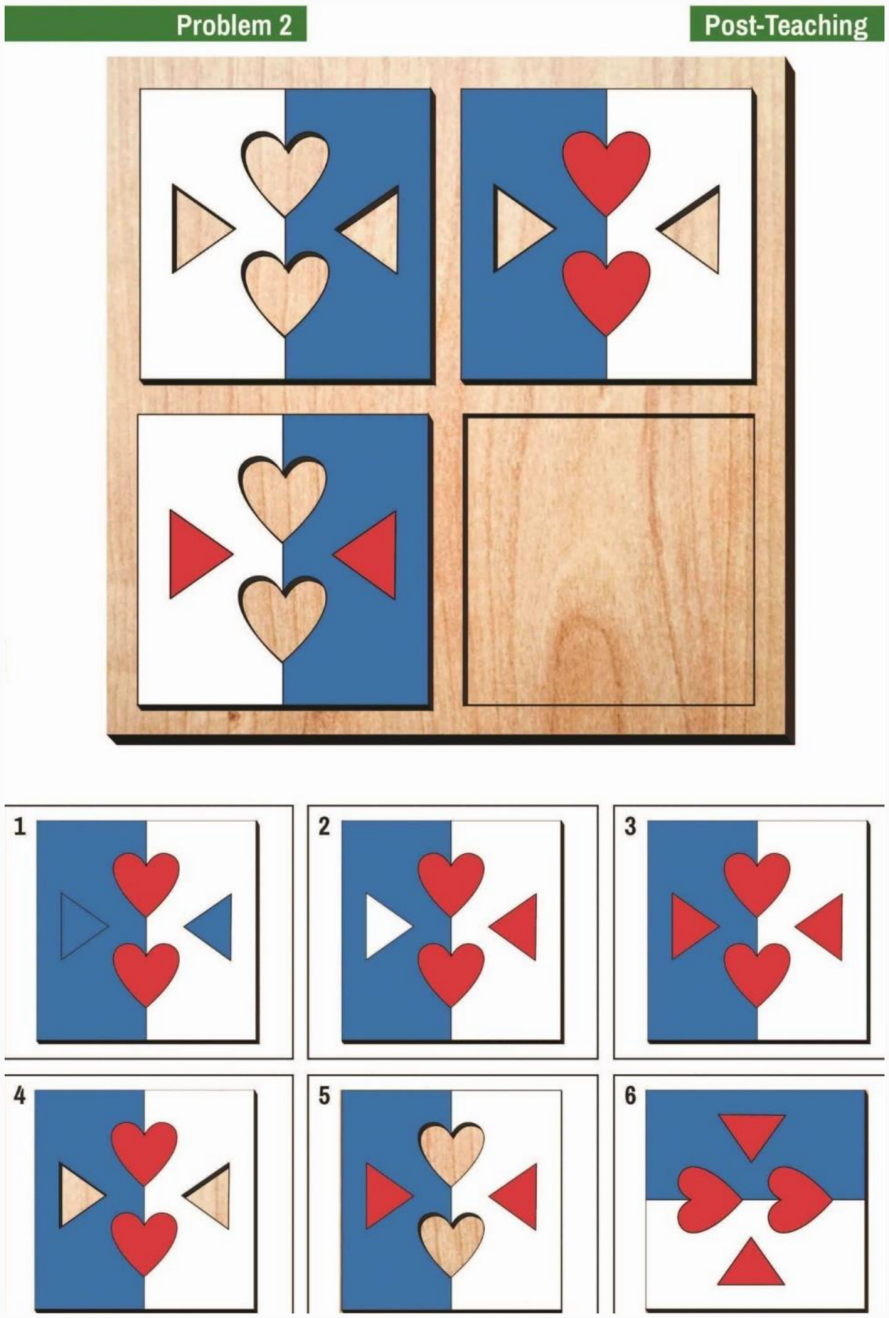
Example of an easy item from the Analogical Modifiability Puzzle Test (AMPT) (by permission of the author).

The AMPT (Tzuriel, 2021a,b, 2022) is a DA measure of cognitive modifiability in the domain of analogical thinking. The AMPT contains three parallel sets of problems, each containing 12 items. The AMPT can be administered either in the *Construction* analogies or *Closed* analogies version (Tzuriel, 2022). In the current study, the Closed analogies version was applied. Before the start of the test, children are presented with two introductory problems aimed at familiarizing the children with the dimensions of AMPT (i.e., color, shape of puzzle parts) and the rules of problem-solving. The items are arranged progressively from easy to difficult problems. In the Closed analogies version, *which is used in the current study*, the child is presented at the top of the page with three sections of the analogy (see Figure 1), and the child must choose the fourth section, from six answers, of which five are distractors. To solve the problem and complete the puzzle, the child must consider the changes from the left to the right section and from the top to the bottom section. The correct solution requires considering changes in the color and position of the panels and shapes from left to right and from top to bottom.

In Figure 1, the colors of the background panels change from left to right (white and blue to blue and white). The hearts on the left sections that are empty are filled up with red hearts on the right, and the empty triangles on the top are filled up with red triangles on the bottom. The correct solution is number 3 (blue and white panels and red hearts and triangles). Cronbach's alpha reliability coefficients for the pre- and post-teaching phases of the AMPT based on a sample of kindergarten children (*n* = 96) were0.94 and 0.95, respectively (Tzuriel, 2022). Cronbach's alpha coefficients for the Pre- and Post-Teaching phases in the current sample were 0.94 and 0.73, respectively. Previous findings showed that a short-term intervention within a DA procedure using the AMPT improved spatial working memory (SWM) and that the improvement in SWM was even more powerful (η_p_^2^ = 61) than the effect on analogical thinking (η_p_^2^ = 0.40) *per se*, which was the targeted mediation activity (Tzuriel, 2022).

#### Math accuracy and processing strategy test (MAPS)

The MAPS test (Tzuriel, 2024), designed specifically for the current study, is aimed at the examination of both performance accuracy and processing strategy. In each item, the children are presented a math problem and asked to solve it and to write how they solved the problem (processing strategy). The test is composed of 10 items and is aimed at children in the second grade. In each item, the child is asked first to solve a mathematical problem and then to explain the strategy used to reach a solution. The explanation was aimed at verifying that the answer is based on a strategy and not on a technical performance. Separate scores are given to accurate answers and to explanations revealing an efficient strategy. A separate score of 1 was given in each item to an accurate answer and to an efficient processing strategy. Examples of problems are shown in Table 1. The Cronbach's alpha coefficients based on the current sample, for pre- and post-intervention, respectively, were Math Accuracy, 0.82 and 0.77; Math Processing Strategy, 0.84 and 0.83; Math Total, 0.77 and 0.77.

**Table 1:** Examples of problems from the Math Accuracy and Processing Strategy (MAPS) Test.

**Item #**	**Problem**	**Solution**	**Processing strategy: explain how you solved the problem?**
3	Here is a math problem with a solution: 19 + 34 = 53. Please use this solution to solve the following problem without using a computation 29 + 24 =		
5	You are asked to count from 2 to 32 skipping 2 each time. Will the number 87 appear later in the series?		
7	In how much the sum of 64 + 8 is smaller than 64 + 28?		
10	Ron is 35 years old, and Dan is 16 years old. How old is Tammy if we know that the sum of all three ages is 60?		

### Procedure

All children were administered the AMPT individually using the measurement/research format and the MAPS test before and after the intervention phase. Children in the EX group received the mediation with 12 analogy teaching items for 1 h. Mediation was focused on the systematic exploration of task dimensions, the deletion of impossible alternatives, the delay of rapid answer and inhibition of behavior, the comparison of the different options, and the verbal anticipation of the answer. Children in the CN group practiced the teaching items with no mediation for the same amount of time. The test was administered in a quiet room in the school during regular school time. The pre-intervention phase was administered to all participants for 1 h followed by the intervention and post-intervention phases that were given between 1 and 5 days later; both tests were administered on the same day with a small break of 10 min in-between.

## Results

### The effects of teaching analogies on analogical thinking and mathematics performance

**Figure 2:**
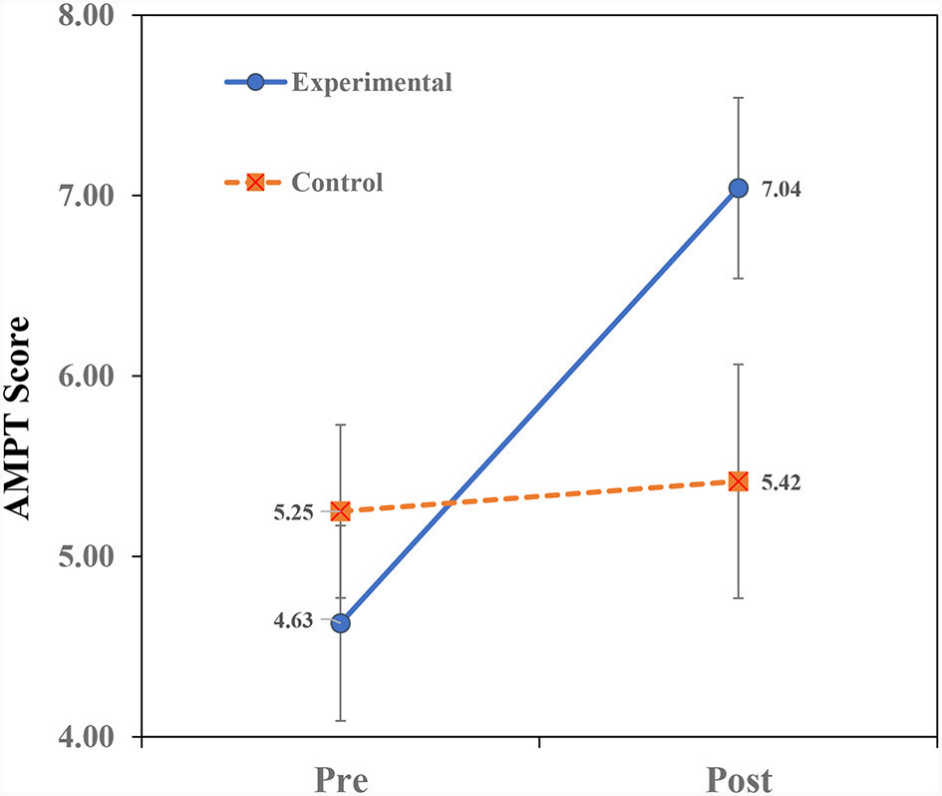
Interaction of treatment × time on AMPT scores.

**Figure 3:**
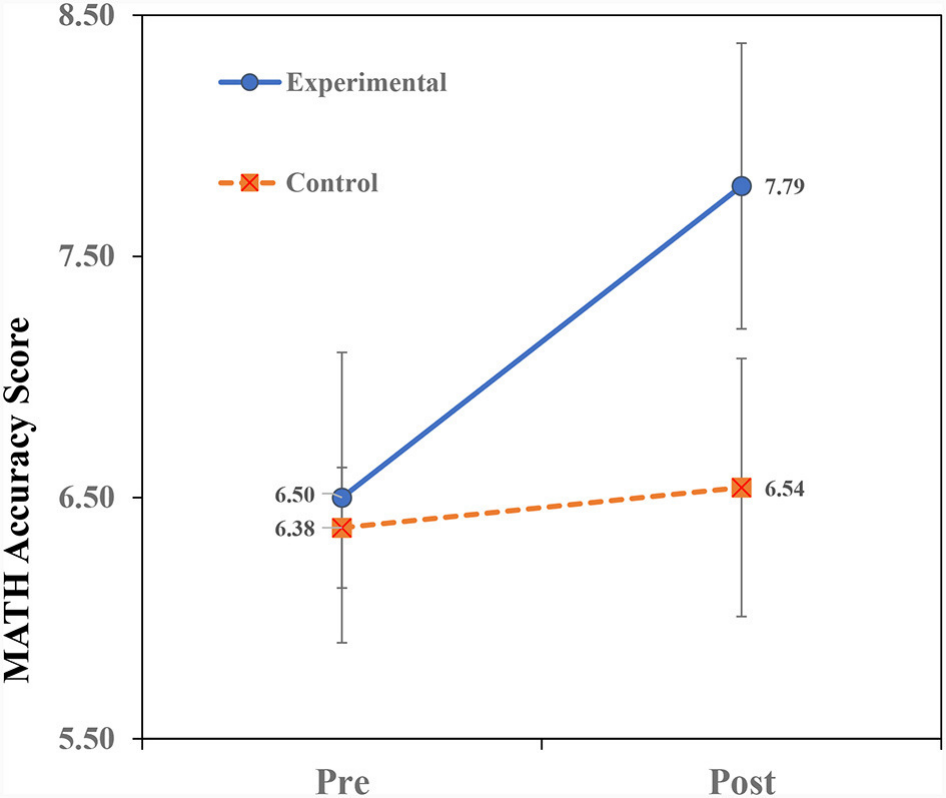
Interaction of treatment × time on math accuracy scores.

**Figure 4:**
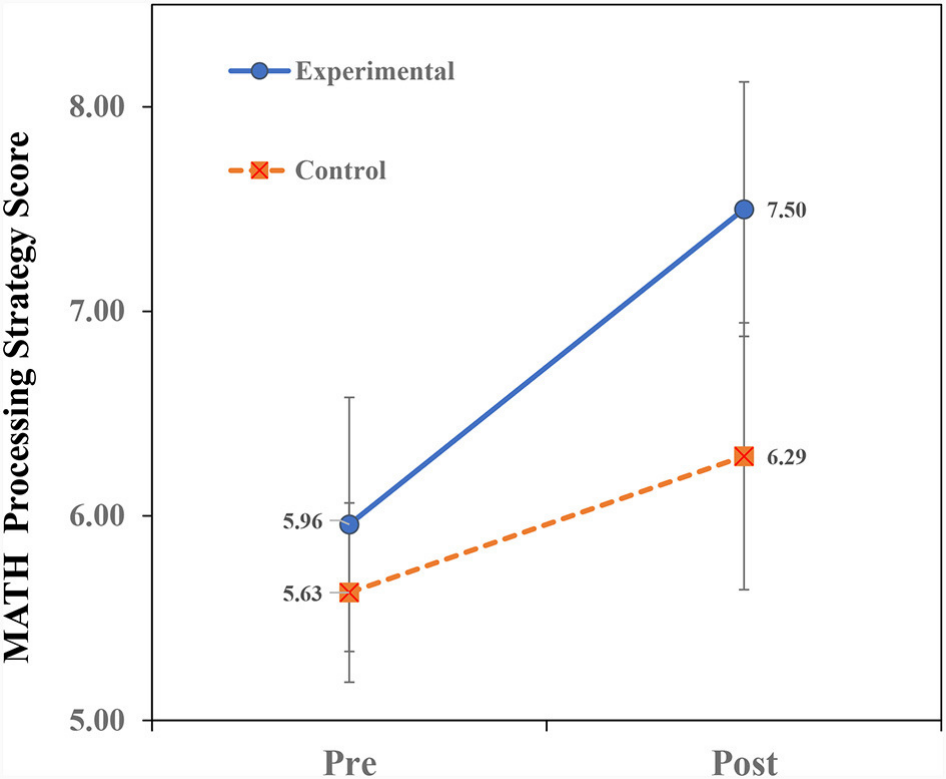
Interaction of treatment × time on math processing strategy scores.

**Figure 5:**
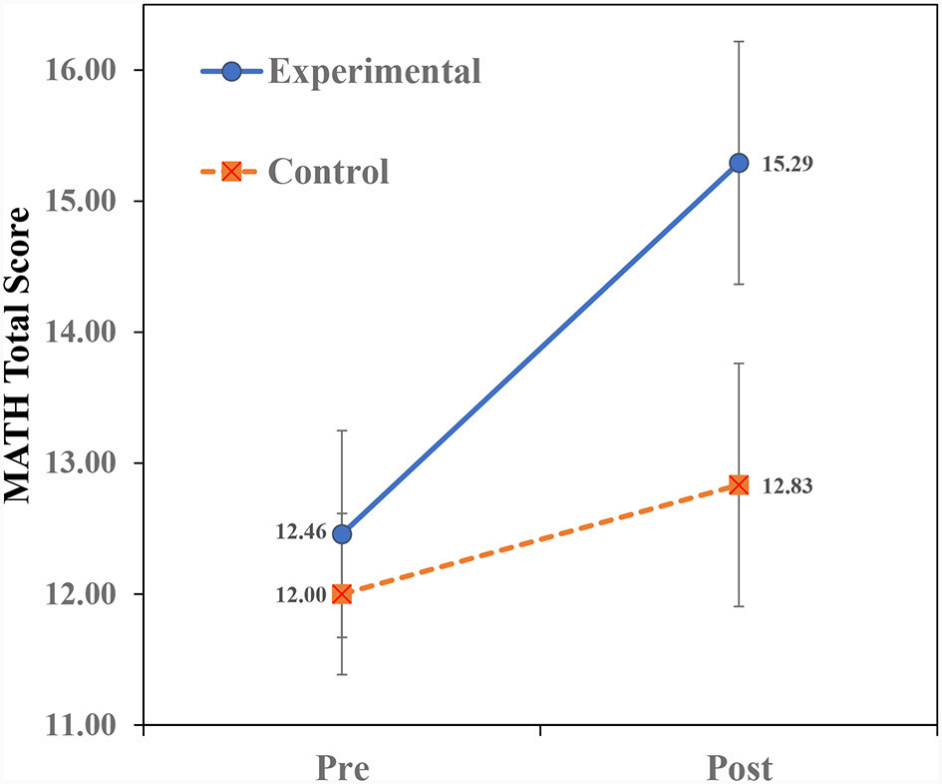
Interaction of treatment × time on math total scores.

Table 2 presents the means and standard deviations of the study's variables. To examine the effect of teaching on analogical thinking modifiability (near transfer), a repeated measures ANOVA of Treatment × Time (2 × 2) for the AMPT scores was conducted. Similarly, repeated measures ANOVAs for the variables (far-transfer) of math were conducted. The ANOVA findings for the AMPT are shown in Table 3 and the ANOVA findings for the math variables are shown in Table 4. Finally, we examined the prediction of Math-Total scores by analogical modifiability using the hierarchical regression procedure. The findings (Tables 3, 4) reveal significant Treatment × Time interactions for all the dependent variables. The interactions are portrayed in Figures 2–**5**. A summary of the simple effects analyses of the interactions is shown in Table 5.

**Table 2:** Means and standard deviations of pre- and post-teaching scores of AMPT, math accuracy, math processing strategy, and math total in the EX and CN groups.

**Test**	**Phase**	**Group**	**M**	**SD**
AMPT	Pre-	EX	4.63	2.65
		CN	5.25	2.45
	Post-	EX	7.04	2.35
		CN	5.42	3.17
Math accuracy	Pre-	EX	6.50	2.95
		CN	6.38	2.90
	Post-	EX	7.79	2.11
		CN	6.54	2.62
Math processing strategy	Pre-	EX	5.96	3.04
		CN	5.63	3.05
	Post-	EX	7.50	2.15
		CN	6.29	3.20
Math total	Pre-	EX	12.46	3.87
		CN	12.00	4.54
	Post-	EX	15.29	3.01
		CN	12.83	4.55

**Table 3:** Repeated measures ANOVA's of AMPT scores by treatment and time.

**Source of variation**	**df**	**MS**	**F**	**η_p_^2^**
Treatment (A)	1	6.00	0.53	0.01
Error	46	11.29		
Time (B)	1	40.04	13.20^***^	0.22
A × B	1	30.38	10.01^**^	0.18
Error	46	3.03

^**^p < 0.01; ^***^p < 0.001.

**Table 4:** Repeated measures ANOVAs of math variables: math accuracy, math processing strategy, and math total by treatment and time.

	**Math accuracy**			**Math processing strategy**			**Math total**		
**Source of** **variation**	**df**	**MS**	**F**	η_p_^2^	**MS**	**F**	η_p_^2^	**MS**	**F**	η_p_^2^
Treatment (A)	1	36.26	4.88^*^	0.10	14.26	0.91	0.02	51.04	1.66	0.04
Error	46	7.44			15.61			30.78		
Time (B)	1	12.76	24.31^***^	0.35	29.26	27.11^***^	0.37	80.67	42.98^***^	0.48
A × B	1	7.59	14.47^***^	0.24	4.59	4.26^*^	0.09	24.00	12.79^***^	0.22
Error	46	0.53			1.08			1.88		

^*^p < 0.05; ^***^p < 0.001.

**Table 5:** Simple effects analyses of treatment × time interactions of AMPT, math accuracy, math processing strategy, and math total variables.

**Test**	**Between group**		**Within groups**	
	**Phase of testing**	***t***_(46)_	**Group**	***t***_(23)_
AMPT	Pre-	0.85	EX	5.86^***^
	Post-	2.02^*^	CN	0.29
Math accuracy	Pre-	0.15	EX	5.13^***^
	Post-	1.82	CN	1.07
Math processing strategy	Pre-	0.38	EX	4.05^***^
	Post-	1.54	CN	3.56^**^
Math total	Pre-	0.38	EX	5.85^***^
	Post-	2.21^*^	CN	2.97^**^

^*^p < 0.05, ^**^p < 0.01, ^***^p < 0.001.

#### Treatment × time interaction on AMPT scores

The significant Treatment × Time interaction (Figure 2) revealed that children in the EX group improved their scores from pre- to post-teaching more than children in the CN group. Simple effects analyses (Table 5) revealed no significant group differences in the pre-teaching phase. However, in the post-teaching phase, children in the EX group outperformed children in the CN group. Within-group analyses revealed a significant improvement from pre- to post-teaching only in the EX group.

#### Treatment × time interaction on math accuracy scores

The significant interaction of Treatment × Time (Figure 3) indicates that children in the EX group showed higher pre- to post-teaching improvement than children in the CN group. Simple effects analyses (Table 5) revealed no significant group differences in the pre- and post-teaching phases. However, within-group analyses showed that only the EX group showed significant pre- to post-teaching improvement.

#### Treatment × time interaction on math processing strategy scores

The significant interaction of Treatment × Time (Figure 4) indicates that children in the EX group showed higher pre- to post-teaching improvement than children in the CN group. The simple effects of between-group analyses (Table 5) revealed no significant group differences in either pre- or post-teaching phases. Within-group analyses showed significant pre- to post-teaching improvement in both the EX and CN groups, though the slope of change was steeper in the EX group.

#### Treatment × time interaction on math total scores

As can be seen in Figure 5, children in the EX group demonstrated higher pre- to post-teaching improvement than children in the CN group. Simple effects between-group analyses (Table 5) revealed no significant group differences in the pre-teaching phase, but in the post-teaching phase, children in the EX group outperformed children in the CN group. Simple effects of within-group analyses showed that both groups revealed pre- to post-teaching improvement, though the EX group demonstrated a much higher improvement.

### Hierarchical regression analysis: prediction of post-teaching MAPS-total by AMPT scores

An intriguing objective of the current study was to assess whether analogical thinking modifiability predicts the modifiability of math performance. According to Hypothesis 3, the modifiability of Total-Math will be predicted by analogical thinking modifiability. In other words, the hypothesis is that a short-term intervention of analogical thinking within a DA procedure will affect not only analogical thinking but will be transferred to math thinking. This hypothesis was evaluated by a three-step hierarchical regression analysis where the predicted variable (criterion) was post-intervention MAPS-Total. In Step 1, the variable of age (in months) was introduced. In Step 2, the variables of pre-teaching variables of MAPS-Total and AMPT were introduced, and in Step 3, the post-teaching variables of MAPS-Total and AMPT were introduced. The modifiability of MAPS-Total score was indicated by the post-teaching score after statistically “washing out” the variance contributed by the MAPS-Total pre-teaching score. Similarly, the modifiability of AMPT score was demonstrated by the post-teaching AMPT score after statistically “washing out” for the variance contributed by the AMPT pre-teaching score. The results, as shown in Table 6, indicate that Age was not a significant predictor of the post-intervention math performance. The variance of Age was negligible, *F*_(1, 46)_ = 0.01, *p* < 0.92, and *R*^2^ = 0.00. In Step 2, however, as expected, the pre-intervention math score was a significant predictor of the post-intervention math score (β = 0.86). The pre-intervention AMPT score was not found as a significant predictor (β = 0.04). The significant prediction of Step 2 added 75% to the prediction of Math-Total score, *F*_(3, 44)_ = 45.63, *p* < 0.0001, Δ*R*^2^ = 0.75, *p* < 0.0001.

**Table 6:** Hierarchical regression analyses of post-teaching math-total score by pre- and post-intervention AMPT scores.

**Step**	**Predicting variables**	**β**	***t***	***p***
1	Age	0.01	0.11	0.92
		*R*^2^ = 0.00, Δ*R*^2^ = 0.00	
		*F*_(1, 46)_ = 0.01, *p* = 0.92	
2	Age	−0.12	−0.1.57	0.12
	Pre-AMTP	0.04	0.53	0.60
	Pre-math-total	0.86	10.42	0.0001
		*R*^2^ = 0.75, Δ*R*^2^ = 0.75, *p* < 0.0001	
		*F*_(3, 44)_ = 45.63, *p* < 0.0001	
3	Age	−0.08	−1.06	0.30
	Pre-AMTP	−0.06	−0.68	0.50
	Pre-math-total	0.81	10.18	0.0001
	Post-AMPT	0.24	2.79	0.008
		*R*^2^ = 0.79, Δ*R*^2^ = 0.04, *p* < 0.008	
		*F*_(4, 43)_ = 41.46, *p* < 0.0001	

In Step 3, after controlling for the variance of the pre-intervention variables, the post-intervention AMPT variable has emerged as a significant predictor (β = 0.24) of post-teaching MAPS-Total (criterion variable), thus confirming Hypothesis 3. The overall prediction of post-intervention MAPS score was significant, *F*_(4, 43)_ = 41.46, *p* < 0001, *R*^2^ = 0.79. The AMPT post-intervention score, as an indicator of analogical modifiability, added significantly to the prediction of post-intervention Math-Total, Δ*R*^2^ = 0.04 *p* < 0.008.

## Discussion

The results of this study support the hypothesis that the mediation of analogical thinking within a DA procedure will significantly improve both analogical thinking (Hypothesis 1, near transfer) and math performance (Hypothesis 2, far transfer). These findings agree with the previous findings that showed an intimate relationship between analogical thinking and math thinking, especially with math processing strategies (Núñez and Lakoff, 2005; Richland et al., 2007; Vamvakoussi, 2017, 2019). However, the results of this study go beyond previous studies by indicating that a short-term intervention in analogical thinking can be transferred to math performance, especially to math processing strategies. Math problem-solving has been conceptualized as a transfer challenge requiring students to apply strategies, skills, and knowledge to novel problems (Fuchs and Fuchs, 2005). This idea is congruent with the schema construction theory according to which students' development of schemas occurs when students group problems into types that require the same solution (e.g., Gick and Holyoak, 1983; Chen, 1999). It should be emphasized that the intervention for solving the AMPT analogies was not limited only to specific strategies of solving analogies *per se* but included mediation for the control of impulsivity, considering two or more sources of information simultaneously, activation of WM, systematic exploratory behavior, and verbal anticipation of a solution. There was no way of separating these cognitive functions from the content aspects of the visual analogies of the AMPT.

It is important to note that math processing has much in common with analogical thinking. For example, the question of “In how much the sum of 64 + 8 is smaller than 64 + 28?” requires a simple analogy—if 8 is smaller than 28 in 20 and 64 is a constant, then 64 + 8 is smaller in 20 than 64 + 28. The analogical processing is a much more efficient way to solve the problem than the meticulous procedure of adding up the sums, calculating each segment, and subtracting one from the other.

The teaching of AMPT items within the DA procedure was found in an earlier study on Grade 1 children to be powerful in improving not only their analogical thinking but was also transferred to significant improvement of spatial WM (Tzuriel, 2022). The improvement in spatial working memory was impressive, as indicated by a more powerful effect (η_p_^2^ = 0.61) than that found on analogical thinking (η_p_^2^ = 0.40), which was the targeted domain of mediation strategies. Moreover, the correlations of AMPT analogies (both pre- and post-teaching) with spatial WM was.47 (*p* < 0.001). It should be emphasized that the contents of the MAPS tasks as well as the spatial WM task were different from the contents of the intervention contents, as represented by the AMPT analogies; thus, the improvement would be attributed to the mediation characteristics of the AMPT. Our findings might pave the way for long-term intervention studies aimed at establishing the effects of analogical thinking intervention on the development of math skills. The question is, of course, to what extent is this transfer durable, especially since the intervention was short within a DA procedure and the well-known phenomenon of fading-out of intervention effects (e.g., Bailey et al., 2020).

The results of the hierarchical regression analyses confirm Hypothesis 3. The results show that the variable of post-teaching AMPT significantly contributed to the prediction of math ability after controlling for the variables of pre-teaching scores of analogical thinking and math. These intriguing findings imply that children who demonstrate analogical thinking modifiability in a DA procedure are also those who show pre- to post-intervention modifiability in mathematical thinking. The meaning of this finding is that a short-term mediation within a DA procedure was transferred to math domain enhancing both math accuracy and math processing strategies. This conclusion, however, should be studied further with different age groups, varying the duration of the intervention and employing different analogy tests. It should be emphasized that the finding of a far transfer after a short-term intervention does not mean that the change is durable. One should bear in mind that the intervention in the current study was within a DA procedure, which provides an important indication for the transfer of intervention of analogical thinking to mathematical processing. For a durable long-lasting effect of the intervention, a more comprehensive intervention and a follow-up assessment should be performed. The finding of a far-transfer of analogical thinking to math processing strategies is of special importance for teaching math to students who experience difficulties in processing math problems. Teaching analogical problem-solving might facilitate math processing even more than the direct teaching of math.

## Limitations

The study has several limitations. First, the study was conducted on a limited sample of 48 Grade 2 students. Despite the impressive findings of far-transfer effects from analogical thinking training to math performance, more research involving bigger samples is required. Second, the MAPS test is composed of only 10 items, which limits the validity of findings. Although there is a separate score for accuracy and for processing strategy (a total of 20) for each item, a much larger scale with more items would help to establish the findings. In fact, in a further unpublished study (Tzuriel, 2024), steps were made to enlarge the number of items in MAPS. The third limitation is related to the lack of recording of the specific mediation strategies used in the intervention phase of the analogies. The assumption is that all examiners use the same mediation strategies. A higher resolution of the specific strategies used and their effect on the transfer to math domain would facilitate not only theoretical construction but also practical implications for teachers.

## Data availability statement

The raw data supporting the conclusions of this article will be made available by the authors, without undue reservation.

## Ethics statement

The study involving human participants was reviewed and approved by the Ethical Committee of Talpiot Academic College. The study was conducted in accordance with the local legislation and institutional requirements. Written informed consent to participate in this study was provided by the parents of children prior to start of data gathering and based on Israel Ministry of Education requirements.

## Author contributions

DT: Conceptualization, Data curation, Formal analysis, Funding acquisition, Investigation, Methodology, Project administration, Resources, Software, Supervision, Validation, Visualization, Writing—original draft, Writing—review & editing.
